# Influences of Thermal Treatment on the Dielectric Performances of Polystyrene Composites Reinforced by Graphene Nanoplatelets

**DOI:** 10.3390/ma10070838

**Published:** 2017-07-21

**Authors:** Benhui Fan, Yu Liu, Delong He, Jinbo Bai

**Affiliations:** Laboratoire Mécanique des Sols, Structures et Matériaux (MSSMat), CNRS UMR 8579, Ecole CentraleSupelec, Université Paris Saclay, Grande Voie des Vignes, 92290 Chatenay-Malabry, France; benhui.fan@ecp.fr (B.F.); yu.liu@ecp.fr (Y.L.)

**Keywords:** dielectric properties, percolation, thermal treatment

## Abstract

Dielectric properties of composites near percolation threshold (*f*_c_) are often sensitive to thermal treatments, and the annealing temperature is usually associated with a polymer’s rheological properties. In this study, the influences of the thermal treatment on dielectric properties are investigated for the polystyrene (PS) matrix composite reinforced by graphene nanoplatelets (GNP) fillers near *f*_c_. It can be found that the thermal treatment can not only increase the dielectric constant, but also decrease the dielectric loss for the PS/GNP composite. This interesting phenomenon possibly happens in the interfacial region of PS/GNP with the thickness about 4–6 nm according to the electron energy-loss spectroscopy (EELS) results. The free volumes around the interface can be easily altered by the movement of polymeric segments after annealing at the glass transition temperature.

## 1. Introduction

Polymer matrix composites are of great interest for advanced functional materials which require the combination of the mechanical flexibility and tunable dielectric properties [1,2,3]. Generally, the dielectric properties of composites depend on the instinct physical/chemical properties of the fillers and the polymer matrix, as well as the interactions between them. In a composite incorporated with conductive fillers, the percolation transition is crucial to the dielectric behavior, which influences the global performances. According to the percolation theory and reported experimental results, the dielectric performances of the composite will change thoroughly when the conductive filler’s volume fraction is approaching a certain value, known as the percolation threshold (*f*_c_). This critical behavior of the dielectric performances can be explained by the micro-capacitor model: in the micro-capacitor structure, two adjacent conductive fillers act as double electrodes and the thin polymer matrix layer in between as dielectric. The large capacitor contributed by these micro-capacitors can be increased as the volume fraction of the conductive fillers until finally a conductive network is formed near *f*_c_. The significant increase in the intensity of the local electric field promotes the migration of the charges on the interfacial regions between fillers and matrix, which causes the dramatic increase in dielectric performance at low frequency by interfacial polarization, also known as Maxwell-Wagner-Sillars (MWS) effects [1,4]. Hence, the dielectric properties of the percolation behavior are always attractive in the field of high dielectric composites [5,6,7,8].

A large variety of factors influence the percolation behavior of composites, such as the physical properties and morphologies of fillers, the nature of polymers, as well as the processing methods used in fabrication [9,10]. Moreover, the dielectric properties of composites near *f*_c_ are often sensitive to the thermal stimulation, as also reported by other papers [1,11,12]. There are various processing methods to prepare polymer matrix composites, including solution casting, melting blending, in situ polymerization, etc., but the most widely-used method for manufacturing is melting blending because of its ease of integration into standard industrial facilities. The procedure of melting blending generally consists of the two following steps: the molten polymer is mixed with fillers by the shear of screws, and afterwards the mixture is shaped by extrusion-injection molding. If the fillers have large aspect ratio, they are unavoidably oriented under flow during injection molding, which makes the dispersion of the conductive fillers inhomogeneous and consequently makes the micro-capacitor network in the composite easily altered by thermal treatments [13,14,15].

However, this sensitivity of the micro-capacitor networks to thermal treatment has also provided an opportunity to further improve dielectric properties of the composite near *f*_c_. In the previous studies, we have already reported the effects of thermal treatments on dielectric properties for a linear semi-crystalline polymer (polyvinylidene fluoride, PVDF) incorporated by carbon nanotube (CNT)/BaTiO_3_ hybrids [16,17]. For the epoxy with a non-linear cross-linked structure, the composite reinforced by CNT/Al_2_O_3_ hybrids has also shown improvements in dielectric properties after multi-cycle thermal treatments at different temperatures which are associated with the glass transition [18]. Actually, besides the linear semi-crystalline polymer and the non-linear cross-linked polymer matrix, the composite of an amorphous polymer matrix may also have potential improvement on dielectric properties after annealing at certain temperatures. The glass transition temperature, T_g_, is a critical temperature for the visco-elastic polymer. It is widely accepted that when the polymer is heated to its T_g_, the molecular chains start to move due to the expansion of free volumes [19,20]. If the composite near *f*_c_ is annealed at T_g_ for long enough time, it is highly possible that dielectric properties may also be altered by the slight change of the free volume around molecular chains in interfacial regions, which enables induction of the shrinkage of the distance between adjacent conductive fillers. Therefore, under this assumption, polystyrene (PS, a widely-used amorphous polymer in the capacitor industry) was selected as the polymer matrix to investigate the influences of the thermal treatment on the dielectric properties of composites. The conductive fillers selected for the study were graphene nanoplatelets (GNP) with a large aspect ratio (the ratio of diameter and thickness). The composites with different volume fractions of GNP were prepared by melting blending and shaped by extrusion-injection. The length of the interfacial region between GNP and PS was quantitatively characterized by electron energy-loss spectroscopy (EELS). Based on the results of dynamic mechanical analysis (DMA), a thermal treatment is conducted for the composites and the dielectric properties of PS/GNP before and after the thermal treatment are well discussed and studied in the following parts.

## 2. Experiments and Characterizations

### 2.1. Materials

**Figure 1:**
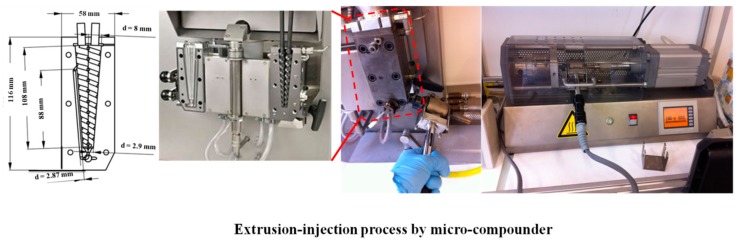
The process of preparing samples by micro-compounder.

GNP (the commercial name is G5) was purchased from Knano Graphene Technology Corporation Limited., Xiamen, China. PS was purchased from Goodfellow, Huntingdon, UK. The powders of PS and G5 with the calculated masses were firstly mixed by melting blending in a twin-screw micro-extruder/compounder (Micro 5cc twin screw compounder, DSM, Xplore Instruments BV, Sittard, The Netherlands) for 30 min at 230 °C with the speed of 60 rpm. The information of the twin-screw extruder is illustrated in the Figure 1. The bone-shaped composite slabs of 1.5 mm thickness were fabricated via the extrusion-injection method (Micro 5cc Injection Molder, DSM, Xplore Instruments BV, Sittard, The Netherlands). The temperatures of the injection nozzle and mould holder were set at 235 °C and 55 °C, respectively.

### 2.2. Characterizations

The morphology of G5 was characterized by a scanning electron microscope (SEM) (Quanta 200 FEG, FEI Company, Hillsboro, OR, USA) at 5 kV. The sample of PS/G5 composite for scanning transmission electron microscopy (STEM) was prepared by focused ion beam (FIB) in a Helios 660 (FEI) Dual Beam FIB-SEM system with a Ga^+^ ion source at 30 kV. The morphologies of G5 and PS/G5-9% composite were characterized by a Titan^3^ G2 with a field-emission gun (XFEG, FEI) operating at an accelerating voltage of 80 kV in STEM mode. EELS tests were carried out on the same equipment and operated at 80 kV in STEM mode. All the images were acquired by high-angle annular dark-field detector. The energy resolution measured with EELS is 1.4 eV. Digital images and energy-loss spectra were captured using an Enfinium ER filter with a dispersion of 0.1 eV/channel. Obtaining spectra in the STEM mode using a camera length of 115 mm and a spectrometer entrance aperture diameter of 5 mm provided a collection semi-angle (β) of 14.7 mrad. Convergence angle (α) was about 16.7 mrad. Further spectral processing operations were conducted using digital micrograph (DM, Gatan, CA, USA) software [21]. LabRAM Raman Spectrometer (LabRAM HORIBA Jobin Yvon, Edison, NJ, USA) excited by the 633 nm coherent line of a He:Ne laser was used to determine the carbon structure of the samples at room temperature. Dynamic mechanical analysis (DMA) was conducted in the tension mode by Netzsch DMA 242C (NETZSCH, Selb, Germany). The measurement was conducted from 25 °C to 150 °C at 1 Hz with a heating rate of 2 °C/min. The size of the specimen was 20 mm × 5 mm × 1.5 mm. The loss tangent for storage modulus is defined as $\tan\theta =\frac{E^{\prime \prime }}{E^{\prime }}$, where *E*’ and *E*” corresponding to the real and imaginary parts of the dynamical (complex) storage modulus E*, respectively. The dielectric properties of composites were characterized as a function of frequency (100 to 10^6^ Hz) by an impedance analyzer (Solartron 1260, Solartron, Hampshire, UK) at room temperature (25 °C). Before the measurement, the silver paste was applied on both sides of each sample for easy contacting. The tested sample was considered as a plane capacitor and described by a parallel resistor-capacitor (RC) circuit system. The complex dielectric permittivity (ε^*^) is calculated as $\varepsilon^{*}=\varepsilon^{\prime }-j\varepsilon^{\prime \prime }$, where ε’ and ε” correspond to the real and imaginary parts of the complex ε*, respectively. ω=2πf is the angular frequency, and $j={\left(-1\right)}^{\frac{1}{2}}$. The dielectric loss tangent (tan δ) is defined as $\tan\delta =\frac{\varepsilon^{\prime \prime }}{\varepsilon^{\prime }}$.

## 3. Results and Discussion

### 3.1. The Morphology and Structure of G5

**Figure 2:**
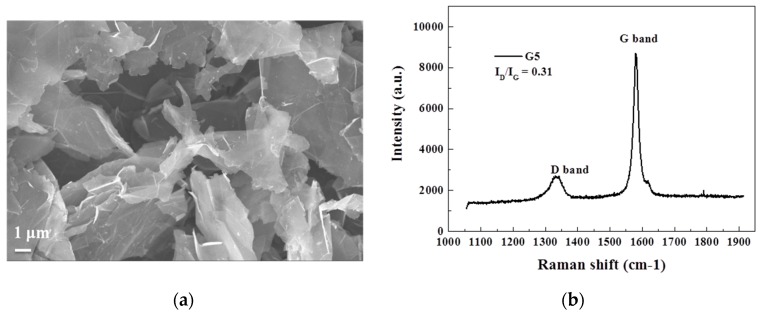
(**a**) SEM image and (**b**) Raman spectrum for G5 powder.

The morphology and the physical properties for G5 were characterized by SEM and Raman spectra, which are presented in Figure 2a,b, respectively. The semi-transparent layer in the SEM image of Figure 2a indicates an extremely thin thickness of G5, and the diameter of a layer was as large as several micrometers. The Raman spectra in Figure 2b illustrate a weak disorder-induced D-band at 1350 cm^−1^ and an incisive graphite-like G band at 1580 cm^−1^ [22]. The ratio between the intensity of two peaks, D and G, (I_D_/I_G_) was employed to roughly estimate the graphitization rate in G5’s structure, and the value is as low as 0.31 which infers few defects in its few-layer structure.

### 3.2. Dielectric Properties for PS/G5 Composites with Different Volume Fractions

**Figure 3:**
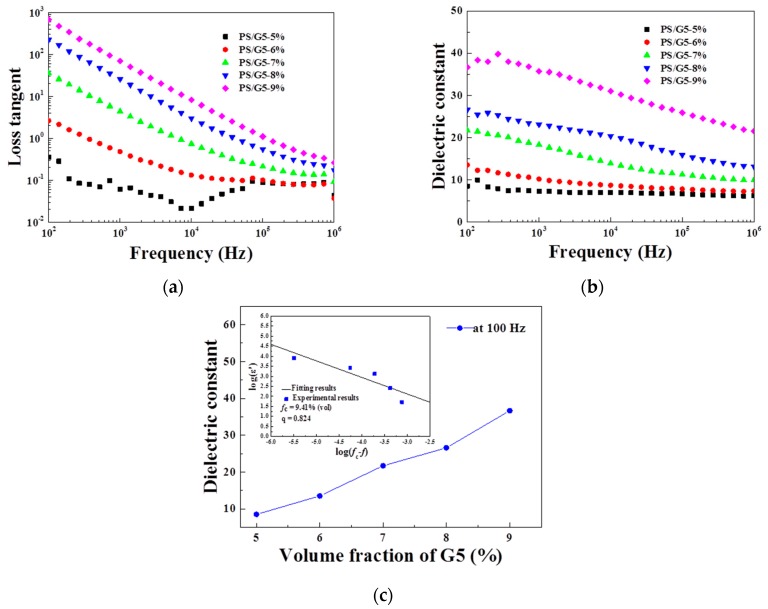
Frequency dependence of dielectric properties for polystyrene/graphene nanoplatelets (PS/G5) composites with different filler volume fractions: (**a**) dielectric loss; (**b**) dielectric constant; and (**c**) the best linear fits for ε’ at 100 Hz for PS/G5 composites with different volume fractions by the percolation theory shown in Equation (1).

As shown in Figure 3a,b, it can be found that tan δ and ε’ of the composites show two features: the dependence of both volume fraction and frequency on dielectric properties, which can be explained by different polarization mechanisms. On one hand, for the phenomenon of volume fraction dependence, the percolation behavior for PS/G5 composites needs to be considered first. The linear fitting by Equation (1) [4] was employed to calculate the theoretical *f*_c_ for the composites, and the calculated result is presented in Figure 3c:

(1) $$\varepsilon_{\mathrm{eff}}\propto \varepsilon_{m}|f_{G5}-f_{c}{|}^{-q},\;\mathrm{for}\;f_{G5}<f_{c}$$

where ε_m_ and ε_eff_ are the dielectric constants for the matrix and composite, respectively; *f*_G5_ is the volume fraction of G5. The results of linear fitting for ε’ at 100 Hz of PS/G5 give q as 0.824, which agrees with the universal value (in the range from 0.8 to 1) and *f*_c_ as 9.41%. According to the percolation’s theory, G5 in the PS matrix can be treated as a micro-capacitor with core–shell structure in the composite system where the impenetrable hard cores represent G5 and the thickness of the penetrable soft shells is related to the tunneling distance [1,4,23]. The two adjacent G5 can be considered to connect via the tunneling effect that relies on the average mutual distance between two adjacent G5. The average mutual distance is dominated by *f*_G5_, and usually the higher the *f*_G5_ is, the smaller the average mutual distance of G5 is in the composite. When *f*_G5_ is much lower than *f*_c_ (as is the case of PS/G5-5%), the network of the micro-capacitor fails to be well-formed, and consequently the interfacial polarization by tunneling effect are not strongly aroused enough by the accumulated charges at interfacial regions between G5 and PS, which makes tan δ and ε’ maintain low values. However, as *f*_G5_ increases gradually, the formation of the micro-capacitor network is gradual in the composite, especially when *f*_G5_ is approaching *f*_c_ (as is the case of PS/G5-9%), the migration of charges at the interfaces is promoted by the significant increase in the intensity of the local electric which strengthens the interfacial polarization and dramatically increases ε’ and tan δ at low frequency. Hence, the dielectric properties of the composites show the volume fraction dependence and ε’ of PS/G5-9% is observed as the highest at low frequency. On the other hand, the frequency dependence of dielectric properties of the composite at high frequency is attributed to different relaxation times in different components of the composites, which is known as the dipolar polarization [1]. In the case of dielectric properties at high frequency, as *f*_G5_ increases, the increase of tan δ and ε’ is less significant compared with the values at low frequency because the interfacial polarization contributes less at high frequency, but the improvement in dielectric properties for the composites with high *f*_G5_ can still be found in Figure 3a,b. The increase of ε’ and tan δ for the composites with higher *f*_G5_ infers that the content of fillers will influence the interaction between G5 and PS by changing the movement of PS segments, which may strengthen the dipolar polarization. However, the dipolar polarization will not arouse a huge increase of tan δ for the composite. Therefore, the large increase of ε’ and tan δ at low frequency for the composite near *f*_c_ is caused by the interfacial polarization, while the increase of dielectric properties at high frequency results from a stronger dipole polarization of matrix due to the improvement of interactions between G5 and PS.

### 3.3. Morphology for PS/G5-9%

**Figure 4:**
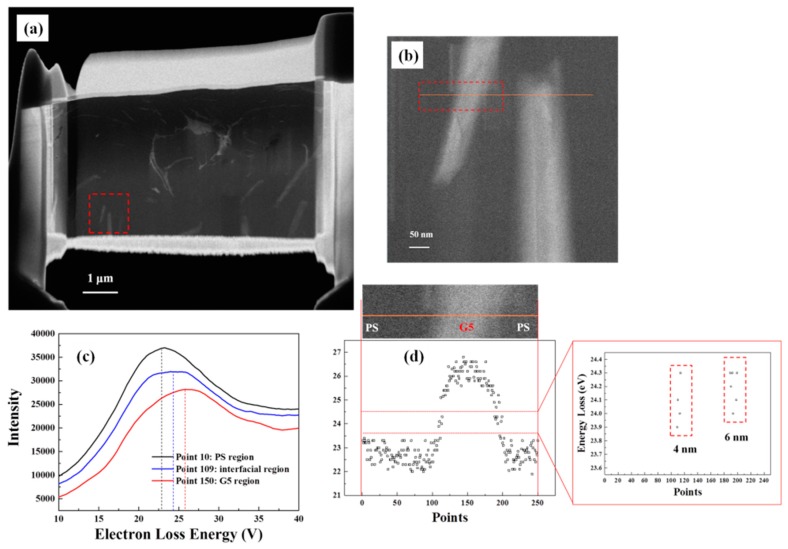
(**a**) The fracture image by scanning transmission electron microscopy (STEM) for PS/G5-9%; (**b**) Higher magnification for the red area in (**a**); (**c**) The energy loss spectra of C-K edges for three points which stand for three regions in the orange line of (**b**); (**d**) Peak positions extracted from electron energy-loss spectroscopy (EELS) acquired from image (**b**), shown as a function of the acquisition order from point 1 to point 250.

Figure 4a shows the laminate of PS/G5-9% prepared by FIB, and it can be found that the G5 (the grey sheets) is well dispersed in PS matrix. In the image of Figure 4b with higher magnification, the interfacial regions around both sides of a single G5 sheet can be well determined, and an orange line-scan of low-energy loss spectra with 500 nm interpreted by 500 points are displayed and the peak points of the first 250 spectra for the left G5 part are extracted and plotted in Figure 4c. According to related research works [21,24,25], the low-energy loss spectrum gives the valence plasmon energy, which could be used to further determine the mass density. For polyaromatic solids, the low loss area exhibits two main plasmon peaks, which are ascribed to excitation of π and π + σ electrons. The π + σ plasmon peaks of probe-points 10, 109, and 150 are 23.2, 24.1, and 26.2 eV, respectively. It is reported that polyaromatic carbon materials of amorphous carbon and graphite show π + σ peak at about 23.5 and 27.5 eV, respectively [25]. Thus, the point of 10 and the point of 150 are in the regions of PS and G5, and the points of 109 can be viewed as the interfacial regions between PS and G5. According to the electron loss energy in Figure 4d, the length of the regions with the energy loss around 24 eV can be viewed as the range of the interface and the values of two interfacial regions are 4 nm and 6 nm, respectively.

### 3.4. Dynamic Mechanical Analysis for PS/G5-9%

**Figure 5:**
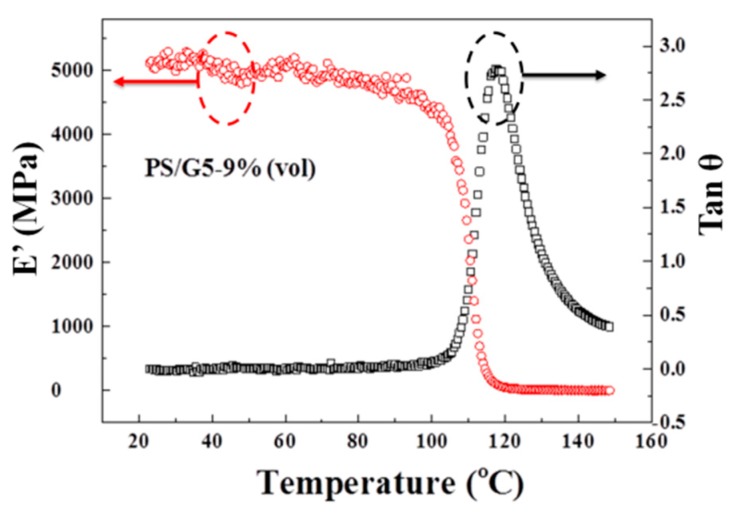
ε’ and tan θ of the dynamic mechanical analysis (DMA) measurement for PS/G5-9% from 25 °C to 150 °C at 1 Hz.

**Figure 6:**
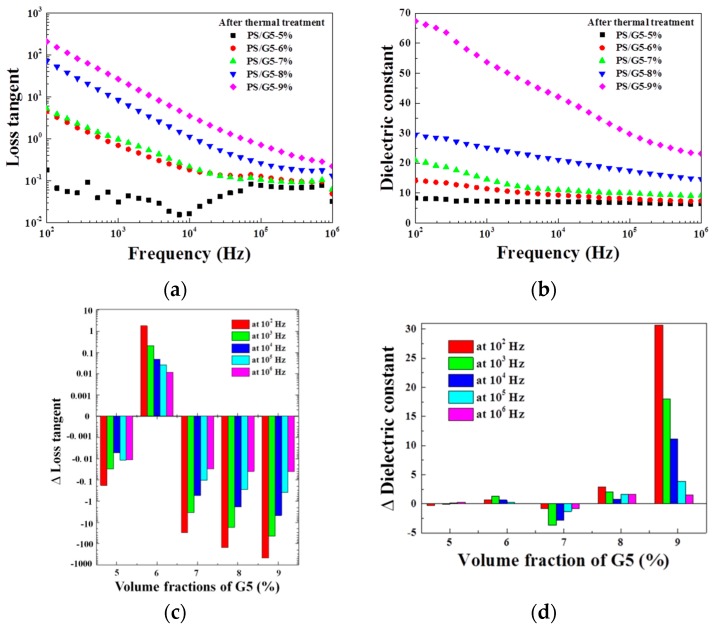
Frequency dependence of dielectric properties for PS/G5 composites series after the thermal treatment: (**a**) for tan δ and (**b**) for ε’; (**c**,**d**) are the differences (∆) of the values of tan δ and ε’ between before and after the thermal treatment at five frequencies, respectively.

We have mentioned that the dielectric properties of composites approaching *f*_c_ can be sensitive to the thermal treatment at a certain temperature. Thus, the effects of the thermal treatment will be investigated in the following parts. DMA is used to detect the rheological behavior for PS/G5-9% from 25 to 150 °C, and the obtained results are plotted in Figure 5. It is widely accepted that polymer’s T_g_ is thought to be is a transitional temperature at which the segment’s movements start, and which corresponds to the temperature of loss tangent’s peak (tan θ). Hence, in Figure 5, 120 °C is regarded as T_g_ for PS/G5-9%. Thus, 120 °C was selected as the temperature for the thermal treatment, and the process of the treatment was as follows: the samples with different *f*_G5_ were annealed at 120 °C for 1 h in the oven and then taken out. After cooling naturally to room temperature, the dielectric properties were measured again. The frequency-dependent dielectric properties including tan δ and ε’ are presented in Figure 6a,b. The differences between before and after thermal treatment of tan δ and ε’ at five frequencies are also presented in Figure 6c,d, respectively.

As presented in Figure 6, it can be found that after thermal treatment, with the exception of PS/G5-9%, the values of ε’ for other four composites with lower *f*_G5_ did not have obvious changes, but the values of tan δ decreased (except PS/G5-6%)—especially the values at low frequency. As aforementioned, the dielectric loss results from the conduction loss and polarization loss of space charges [4,8]. The annealing treatment at T_g_ and the followed cooling process may alter the states of polymeric segments and the free volume in interfacial regions that modifies the interaction of G5 and PS. Furthermore, as shown by EELS, the length of the interfacial region is around 4–6 nm, and such a short distance is possibly sensitive to the thermal treatment. Hence, an optimized micro-capacitor network in the composite after the thermal treatment will help to reduce the dielectric loss. Moreover, the increase of ε’ in the sample of PS/G5-9% indicates a stronger polarization happening in the composite near *f*_c_. This decrease of tan δ resulting from the change of the free volume in interfacial regions by segment’s movements may be more subtle for achieving globally homogeneous dispersion of G5 in the composite which is different from the shrinkage of the average distance of conductive fillers by simply adding more contents of G5 in the matrix. Although the glass transition can similarly alter the movements of polymeric segments and free volumes in composites with lower *f*_G5_, the original average distances of G5 in these four samples may be too large to induce the tunneling effect, and even after the thermal treatment, the average distances in these samples are still not approaching the critical value. Therefore, the thermal treatment can reduce the tan δ for PS/G5 composites and meanwhile improves the dielectric properties on the composite near *f*_c_ where the micro-capacitor network is sensitive to the external stimulation. This influence possibly occurs in the interfacial regions, which can not only increase ε’ at low frequency but also reduce tan δ.

## 4. Conclusions

The dielectric properties of G5-reinforced PS composites can be further improved by thermal treatment, which is associated with the T_g_ of the composite. After the thermal treatment, a modified micro-capacitor network can be formed in the composite which helps to reduce the tan δ of the composite. Moreover, for the composite near *f*_c_, the thermal treatment can increase ε’ but decrease tan δ. This interesting phenomenon possibly happens in the interfacial region of G5/PS with a thickness of about 4–6 nm detected by EELS where the free volume can be easily altered by the movements of polymeric segments after annealing at T_g_ for long enough time.
